# Supplementary material for: Community health workers’ barriers and facilitators to use a novel mHealth tool for motivational interviewing to improve adherence to care among youth living with HIV in rural Nepal
Source: PLOS Glob Public Health. 2024 Jul 11;4(7):e0002911. doi: 10.1371/journal.pgph.0002911 (PMC11238997; doi:10.1371/journal.pgph.0002911)
Supplement: Attachment — Submitted filename: Response to the reviewers.docx [file pgph.0002911.s001.docx]

Dear Editors,

Thank you for the review of our manuscript. We appreciate the comments and suggestions from the reviewers. Below, we note the specific comments made by the reviewers with our responses in bold. All changes made to the manuscript are marked, as suggested. Please note that the line numbers here refer to the manuscript with track change version.

Journal Requirements

1. In the ethics statement in the Methods, you have specified that verbal consent was obtained. Please provide additional details regarding how this consent was documented and witnessed, and state whether this was approved by the IRB.

**We have updated the Ethics Approval section with details on how the consent was documented. It now reads:**

***“The qualitative data collection was done amidst the pandemic and therefore, we obtained verbal informed consent from all individual participants. The Ethical Review Board of Nepal provided approval for the same. To establish trust during virtual data collection, the interviewer made introductory calls with the participants and explained the purpose of the study and their role in it, following which interviews were conducted at the convenience of the participants. Before starting the interview, the interviewer read the consent script and obtained verbal informed consent from the participants. Since the interviews took place over the phone, the audio recording started only after the participant’s consent, which was acknowledged by the interviewer and participant at the beginning of the interview.”* [Line 189-197; added line: 195-197]**

2. Please send a completed 'Competing Interests' statement, including any COIs declared by your co-authors. If you have no competing interests to declare, please state "The authors have declared that no competing interests exist". Otherwise please declare all competing interests beginning with the statement "I have read the journal's policy and the authors of this manuscript have the following competing interests:"

**We have added the following statement:**

**I have read the journal’s policy and the authors of this manuscript have the following competing interests:**

**Author Bibhav Acharya is currently serving as an Editor at PLOS Global Public Health. Rest of the authors have declared that no competing interests exist.**

Reviewers’ Comments:

Reviewer #1: Overall, this is an interesting and useful paper, but it can be further improved by adding some methodological and intervention-related details and justifications regarding the analysis approach. I think the approach seemingly taken makes sense for this kind of intervention-focused study, but the reader currently has to fill in some blanks about the qualitative methods.

The article is well-written, provides a useful background, and most parts of the process are well explained and justified. You could also mention this predominantly being a pragmatic/directed/descriptive qualitative study. You have not really mentioned in the abstract either what kind of study this is – “results of semi-structured interviews” does not tell the reader very much yet. Did you use some kind of qualitative reporting guidelines, e.g., SRQR? That might help to ensure all crucial details are included (many of which are, such as the role/relationship of the interviewer etc.).

**Thank you very much for your encouragement and suggestions on the manuscript**. **In the abstract, we have edited the line to now read as, “In this descriptive qualitative study, we share the results from semi-structured interviews.......” [Line 63-64].**

Some more details on the analysis approach in particular would be helpful. Did you use any specific analysis methods that you could cite (e.g., what kind of thematic analysis?), or if not, could you explain more about the approach you developed yourselves? E.g., what guided coding and theme development – was it very data-driven/deductive or mainly informed by the “search” for barriers and facilitators? Rather state this explicitly than having the reader guessing based on your analytic output. Remember, if your interview guides mainly covered barriers and facilitators, then it will not sound coherent to describe the analysis as open-ended and inductive or similar while reporting on mainly barriers and facilitators. Similarly, what significance does it have that you “single-coded” – does this mean one person did all the coding once? Clarify and justify.

**We have added further details in the analysis section of the manuscript. It now reads:**

***“******Coding was based on both a priori code book on potential barriers and facilitators, while allowing new codes to emerge, mainly in response to questions that asked about general reactions to the experiences of using the tool. We coded the data using Dedoose, a qualitative data management software. RK, the interviewer, coded all the data. After the coding was done, based on the similarity in the patterns of the codes, different themes and sub-themes were derived from the data. The themes touched upon perception of MI, experiences of using MI, changes in MI skills, experiences of stigma, supervision experiences, usefulness of MI, and barriers faced by CHWs and facilitators for them to use COMMIT+. This paper focuses only on the barriers and facilitators for CHWs to use COMMIT+.”* [Line 173-181]**

**We have also edited the Tools and Data Collection to add clarity about the interview tools. It now reads:**

***“The study team members developed and finalized two semi-structured guides to conduct the interviews with CHWs and CHNs using similar approaches: beginning with open-ended questions followed by probes regarding their*** ***experiences of receiving training, understanding of MI, experiences in learning and using COMMIT+, receiving and providing supervision, and responses of clients. We asked both CHWs and CHNs about general facilitators and barriers experienced by CHWs while engaging with the clients to deliver MI.”* [Line 149-154]**

When reading the discussion, I wonder if providing a bit more information about the COMMIT+ tool itself early on in the article (e.g., how was it used by CHWs/CHNs in practice, example prompts and a bit more description about what the CHWs and CHNs wanted it to include in the HCD/co-design phase, were they keen on the recording then for example?) would also help contextualise the findings and discussion? I assume this has been published in detail elsewhere, but some kind of text box/figure/similar or just a few more sentences in the main text would be beneficial here too.

**We have added the following information about the COMMIT+ tool, and the description now reads as:**

***“CHWs often lack this kind of ongoing supervision because they travel from one client’s home to another without meeting their supervisor and COMMIT+ was designed to overcome this challenge.*** ***Detailed description of the tool and the co-design process will be described in a forthcoming manuscript. Briefly, the tool was used by CHWs while counselling the clients. The tool guides the CHWs to engage with clients, prompting the CHWs to apply appropriate MI skills depending on the clients’ stages of change. For example, if the client is in pre-contemplative phase, the tool prompts the CHWs to use “sustain talk” and ask open-ended questions to the clients. The tool has a recording feature, which allows the conversation between CHWs and the clients to be recorded, upon consent, for supervision purposes.”* [Line 104-111]**

Lastly, I am pleased to see an example of successfully (even if not without challenges) introducing new responsibilities/approaches to CHWs’ work, as this is often done without consultation and with the expectation that volunteer or low-paid CHWs will just keep taking on more and more tasks (which understandably tends to fail).

**Thank you. We have edited a line to highlight the CHWs are trained, paid, and supervised. The line now reads:**

***CHWs in the study site are local women who have completed 10th grade, and are trained, paid, and supervised.* [Line 125-126]**

Reviewer #2: A very timely and pertinent paper targeting the most vulnerable population i.e the youth who are on HIV care. The paper is well written but there is need to follow the COREQ reporting guidelines for qualitative work especially on domains 1 (research team and reflexivity) and domain 3 (analysis and findings).

**Thank you for the positive comments. Thank you for pointing out the importance of using COREQ guidelines. We agree that we should cover all domains. We have added domain 3 under Reviewer 1’s responses above. For domain 1, we have edited the tools and data collection section as follows:**

***“RK, MSW, who was not part of the MI training and supervision for CHWs and CHNs, conducted the interviews…………The interviewer, RK, is a female, native Nepali speaker, and did not have a supervisory role for the CHWs or the CHNs.*** ***She was employed as a Qualitative Research Manager by the research team. The participants were familiar with her as she had attended their meetings, interacted with them, and accompanied some of them for home visits to learn about the CHW program.”* [Line 157, 162-166]**

Also clarify if the 5 CHNs interviewed twice if there was a difference and if the results for both interviews were used for this manuscript or not.

**The results of both interviews with the CHNs are used for this manuscript. We have highlighted the changes such as the CHNs feeling more comfortable with the supervision process as they spent more time doing so, while receiving support from the study team. It now reads:**

***“In the first round of interviews, a few supervisors shared they did not feel entirely confident about supervising CHWs. In the second round, all supervisors noted that they felt more comfortable in supervising CHWs based on the supervision guidelines and via the support of the study psychologist, with whom they regularly discussed their challenges.”* [Line 307-309]**
