# Supplementary material for: Community health workers’ barriers and facilitators to use a novel mHealth tool for motivational interviewing to improve adherence to care among youth living with HIV in rural Nepal
Source: PLOS Glob Public Health. 2024 Jul 11;4(7):e0002911. doi: 10.1371/journal.pgph.0002911 (PMC11238997; doi:10.1371/journal.pgph.0002911)
Supplement: Attachment — Submitted filename: Response to the reviewers.docx [file pgph.0002911.s002.docx]

Dear Editors,

Thank you for the review of our manuscript. We appreciate the comments and suggestions from the reviewers. Below, we note the specific comments made by the reviewers with our responses in bold. All changes made to the manuscript are marked, as suggested. Please note that the line numbers here refer to the manuscript with track change version.

Journal Requirements:

1. Please review your reference list to ensure that it is complete and correct. If you have cited papers that have been retracted, please include the rationale for doing so in the manuscript text, or remove these references and replace them with relevant current references. Any changes to the reference list should be mentioned in the rebuttal letter that accompanies your revised manuscript. If you need to cite a retracted article, indicate the article’s retracted status in the References list and also include a citation and full reference for the retraction notice.

**We have checked the reference list, and it is complete and correct. No changes have been made in the revised manuscript.**

2. Please send a completed 'Competing Interests' statement, including any COIs declared by your co-authors. If you have no competing interests to declare, please state "The authors have declared that no competing interests exist". Otherwise please declare all competing interests beginning with the statement "I have read the journal's policy and the authors of this manuscript have the following competing interests:"

**We have added the following statement:**

***I have read the journal’s policy and the authors of this manuscript have the following competing interests:***

***Author Bibhav Acharya is currently serving as an Academic Editor at PLOS Global Public Health. Rest of the authors have declared that no competing interests exist.***

**Comments to the Author**

1. If the authors have adequately addressed your comments raised in a previous round of review and you feel that this manuscript is now acceptable for publication, you may indicate that here to bypass the “Comments to the Author” section, enter your conflict of interest statement in the “Confidential to Editor” section, and submit your "Accept" recommendation.

Reviewer #1: All comments have been addressed

Reviewer #2: All comments have been addressed

2. Does this manuscript meet PLOS Global Public Health’s [publication criteria](https://journals.plos.org/globalpublichealth/s/criteria-for-publication)? Is the manuscript technically sound, and do the data support the conclusions? The manuscript must describe methodologically and ethically rigorous research with conclusions that are appropriately drawn based on the data presented.

Reviewer #1: Yes

Reviewer #2: Yes

3. Has the statistical analysis been performed appropriately and rigorously?

Reviewer #1: N/A

Reviewer #2: N/A

4. Have the authors made all data underlying the findings in their manuscript fully available (please refer to the Data Availability Statement at the start of the manuscript PDF file)?

The [PLOS Data policy](https://journals.plos.org/globalpublichealth/s/data-availability) requires authors to make all data underlying the findings described in their manuscript fully available without restriction, with rare exception. The data should be provided as part of the manuscript or its supporting information, or deposited to a public repository. For example, in addition to summary statistics, the data points behind means, medians and variance measures should be available. If there are restrictions on publicly sharing data—e.g. participant privacy or use of data from a third party—those must be specified.

Reviewer #1: Yes

Reviewer #2: No

**We had updated our Data Availability Statement as:**

***This study used qualitative data, and quotes from the participants are included within the manuscript. The data contains personal identifiers and sensitive information of the participants and their clients, who are youth living with HIV, and these identifiers are interspersed in the whole transcript. Therefore, the data cannot be shared publicly in compliance with the regulations of the Ethical Review Board of the Nepal Health Research Council. For further information, please write to approval@nhrc.gov.np. For data access, please submit a reasonable request to the Research and Innovation department at Possible by emailing research@possiblehealth.org.***

5. Is the manuscript presented in an intelligible fashion and written in standard English?

PLOS Global Public Health does not copyedit accepted manuscripts, so the language in submitted articles must be clear, correct, and unambiguous. Any typographical or grammatical errors should be corrected at revision, so please note any specific errors here.

Reviewer #1: Yes

Reviewer #2: Yes

6. Review Comments to the Author

Please use the space provided to explain your answers to the questions above. You may also include additional comments for the author, including concerns about dual publication, research ethics, or publication ethics. (Please upload your review as an attachment if it exceeds 20,000 characters)

Reviewer #1: (No Response)

Reviewer #2: I appreciate the authors' thorough response to the initial round of feedback. They have successfully addressed several concerns, including the improved description of the methodology. The discussion section is more robust, but I encourage the authors to delve deeper into the implications of their findings for future research and policy. Consider discussing potential strategies informed by the study. While the limitations are adequately addressed, providing a more nuanced discussion on how these limitations may have influenced the study's outcomes would enhance the manuscript's transparency. I want to acknowledge the authors' diligence in revising the manuscript based on the initial feedback, and I believe that these additional suggestions will contribute to the manuscript's overall strength. Thank you for considering my second review, and I look forward to witnessing the continued development of this valuable contribution to the field.

**Thank you very much for acknowledging our responses and providing further feedback for the discussion section. We have added the following summary to describe implications for future research and policy.**

***“For future research studies, especially among sensitive research populations, it seems important to take cultural contexts into consideration and employ strategies that support community workers to adequately build rapport and trust with research participants, while rolling out digital tools. Certain features of the mHealth tools—the audio recording feature in our case—can be concerning for end users leading to initial resistance but our data has shown that supportive supervision with a non-judgmental learning environment can be effective at overcoming the barriers experienced by end users. However, future studies can assess implementation strategies that will increase the likelihood of initial uptake of novel digital health features.”* [Line 460-467]**

**Similarly, we have edited and updated the following to reflect on limitations and study outcomes, and it reads as:**

***“Although the tool only had high-yield items that CHWs can view while interacting with clients, this finding may indicate the importance of adding the training materials in a different part of the tool that can be used as a reference. It is possible that using this strategy may have increased ease of use of motivational interviewing, and this is something that can be tested in future studies.”* [Line 448-452]**

***“Although we had not emphasized this aspect in our study, the CHWs informally access peer-based learning. As such, our study may have been unable to uncover the impact of such peer-based support. This can be formally incorporated into future studies by building in structures to foster and study peer supervision.”* [Line 456-459]**
